# Preparation and application of Ag plasmon Bi_3_O_4_Cl photocatalyst for removal of emerging contaminants under visible light

**DOI:** 10.3389/fmicb.2023.1210790

**Published:** 2023-06-09

**Authors:** Zeqing Long, Tingting Guo, Chao Chen, Guangming Zhang, Jia Zhu

**Affiliations:** ^1^Department of Public Health and Preventive Medicine, Changzhi Medical College, Changzhi, China; ^2^Heping Hospital Affiliated to Changzhi Medical College, Changzhi, China; ^3^School of Energy & Environmental Engineering, Hebei University of Technology, Tianjin, China; ^4^School of Materials and Environmental Engineering, Shenzhen Polytechnic, Shenzhen, China

**Keywords:** emerging contaminants (ECs), plasmon, Bi_3_O_4_Cl, environmental purification, visible light, biotoxicity

## Abstract

Photocatalytic degradation has been extensively investigated toward the removal emerging contaminants (ECs) from water. In this study, a series of Ag-Bi_3_O_4_Cl plasmon photocatalysts were synthesized through the photo-deposition of metallic Ag on the Bi_3_O_4_Cl surface. The effects of plasmon modification on the catalytic performance of bismuth oxychlorides were analyzed. Ag addition did not alter the morphology of Bi_3_O_4_Cl. With the increasing Ag content, the number of oxygen defects on the catalyst surface first increased and then decreased. Moreover, the surface plasmon resonance effect of Ag suppressed the recombination of electron–hole pairs, promoting the migration and separation of photocarriers and improving the light absorption efficiency. However, the addition of excessive Ag reduced the number of active sites on the Bi_3_O_4_Cl surface, hindering the catalytic degradation of pollutants. The optimal Ag-Bi_3_O_4_Cl photocatalyst (Ag ratio: 0.025; solution pH: 9; dosage: 0.8 g/L) achieved 93.8 and 94.9% removal of ciprofloxacin and tetrabromobisphenol A, respectively. The physicochemical and photoelectric properties of Ag-Bi_3_O_4_Cl were determined through various characterization techniques. This study demonstrates that introducing metallic Ag alters the electron transfer path of the catalyst, reduces the recombination rate of electron–hole pairs, and effectively improves the catalytic efficiency of Bi_3_O_4_Cl. Furthermore, the pathways of ciprofloxacin degradation products and their biotoxicity were revealed.

## 1. Introduction

Pharmaceutical and personal care products (PPCPs) and endocrine-disrupting chemicals (EDCs) are representative of emerging contaminants (ECs) that possess a bioaccumulation tendency and non-degradability (Liu et al., 2021; Wang et al., 2021); They are harmful to the nervous system of humans and other organisms, affect the function of the endocrine system, and endanger the reproductive capacity of humans and animals (Zhao et al., 2018). Ciprofloxacin (CIP), as a typical PPCP, has been widely used as a broad-spectrum antibiotic. Tetrabromobisphenol A (TBBPA) is the most industrially consumed flame retardant and considered as an EDC (Law et al., 2006; Klein et al., 2020). The complete removal of these pollutants through conventional water treatment processes is difficult (Morin-Crini et al., 2022). Therefore, there is an urgent need to develop new and efficient processes for the removal of these ECs from water.

Photocatalytic technology has attracted increasing attention in recent years owing to its sustainability and high contaminant mineralization rate (Long et al., 2020a). Previous studies have shown that photocatalytic technology can effectively remove ECs from water (Jiang et al., 2019). However, traditional photocatalysts (such as TiO_2_ and ZnO) have low solar light harvest efficiency and high charge recombination rate, limiting their activities and EC removal performance (Long et al., 2020b). Therefore, developing new and efficient visible-light-driven photocatalysts is necessary.

Recently, photocatalysts based on bismuth oxychlorides (BiOCl, Bi_3_O_4_Cl, Bi_12_O_17_Cl_2_, and Bi_24_O_31_Cl_10_) have been extensively studied (Xinping et al., 2006; Cui et al., 2016; Chang et al., 2017). The distinctive layered structure of bismuth oxychlorides is favorable to form an internal electric field, thereby accelerating electron–hole pair separation and imparting excellent optoelectronic properties (Long et al., 2020c). Researchers have worked extensively on modifying bismuth oxychlorides to further enhance their photocatalytic activities. Plasmon modification is currently one of the most important methods for catalyst modification (Zhang et al., 2019). Its principle is to exploit the plasmon resonance effect of noble metals to form a Schottky barrier on the interface with the catalyst, thus accelerating charge transfer, promoting electron–hole pairs separation, and enhancing light absorption by the catalyst. In addition, the increased current density can heat the metal particles, thereby accelerating the photochemical reaction (Hou et al., 2011). Yu et al. (2013) reported a series of (Rh, Pd, Pt)-BiOCl plasmon photocatalysts exhibiting excellent photocatalytic performance in visible light because the plasmon resonance generated by the noble metal nanoparticles improved the charge separation efficiency through electron capture. Xu et al. (2019) prepared a novel Ag@BiOCl plasmon photocatalyst, wherein the Schottky barrier formed by the BiOCl semiconductor and Ag nanoparticles served as a photoelectron capture center to to separate the photogenerated charges. Hence, the photocatalytic performance of the bismuth-oxychloride-based system was significantly improved through plasmon modification.

According to previous studies, among various bismuth-oxychloride-based photocatalysts, Bi_3_O_4_Cl has a more positive oxidation potential to oxidize pollutants, and its bandgap is suitable for visible-light-driven photocatalysis (Long et al., 2022a). Moreover, Bi_3_O_4_Cl has a desirable oxygen defect concentration to accelerate electron–hole pair separation (Cui et al., 2018). Therefore, Bi_3_O_4_Cl was used as the base material in this study, and relatively low-cost metallic Ag was used for plasmon modification. The performance of the Ag-Bi_3_O_4_Cl photocatalyst toward the removal of CIP and TBBPA was evaluated. A series of characterization techniques to analyze the physicochemical and photoelectric properties of the photocatalyst were conducted in order to reveal the modification and electron migration mechanisms. The results provide insights into the development of plasmon-modified bismuth oxychloride photocatalysts and their application in the removal of ECs from water.

## 2. Experimental

### 2.1. Materials and methods

(Supplementary material).

### 2.2. Synthesis of Ag-Bi_3_O_4_Cl plasmonic photocatalysts

To synthesize the Bi_3_O_4_Cl nanoflakes, KCl (4 mmol) powder and deionized water (60 ml) were added to a 200 ml beaker. Subsequently, Bi(NO_3_)_3_^⋅^5H_2_O (4 mmol) was added, and the mixture was thoroughly stirred for 30 min to obtain a white suspension. The suspension was ultrasonicated for 5 min to achieve a uniform dispersion. Next, a NaOH (1 mol/L) solution was added dropwise, and the pH of the suspension was adjusted to 12.0 upon vigorous stirring. Subsequently, the suspension was transferred to a 100 ml Teflon hydrothermal reactor and maintained at 160°C for 12 h. The precipitate was separated and washed three times with deionized water and ethanol. Finally, Bi_3_O_4_Cl nanoflakes were obtained by drying the product in an air oven at 80°C for 4 h.

To synthesize Ag-Bi_3_O_4_Cl plasmonic photocatalysts, the prepared Bi_3_O_4_Cl nanoflakes (0.736 g, 1 mmol) and deionized water (60 ml) were placed in a beaker and stirred for 30 min. Then, the standard AgNO_3_ standard solution (0.01, 0.025, 0.05, and 0.1 mmol) was added, and the mixture was stirred for 2 h under a 500 W xenon lamp. Subsequently, the product was washed three times with water and alcohol and dried in an oven at 80°C for 4 h. The prepared photocatalysts were denoted as 0.01-Ag-Bi_3_O_4_Cl, 0.025-Ag-Bi_3_O_4_Cl, 0.05-Ag-Bi_3_O_4_Cl, and 0.1-Ag-Bi_3_O_4_Cl, wherein 0.01, 0.025, 0.05, and 0.1 represent the molar concentration of the AgNO_3_ solution.

### 2.3. Characterization

(Supplementary material).

### 2.4. Photocatalytic tests and data analysis

In this study, the catalytic activity of the catalyst was assessed by the photocatalytic decomposition of ECs. A certain amount of photocatalysts and CIP (50 ml; 10 mg/L) or TBBPA (50 ml; 10 mg/L) solution were added to a quartz reactor and sonicated for 30 s. Next, the quartz reactor was placed in a PCX50C Discover photocatalytic reaction system equipped with a 5 W white LED lamp as a visible light source (400 nm ≤ λ ≤ 800 nm, Perfect Light Co., Ltd., Beijing, China).

Before irradiation, the mixture of the photocatalyst and contaminant solution (CIP or TBBPA, 10 mg/L) in the quartz reactor was stirred under dark conditions for 30 min to achieve the adsorption–desorption equilibrium. Then, the light source was turned on, and samples were extracted at specific intervals using a syringe. Next, the extracted solution was filtered through a 0.22 μm nitrocellulose filter to remove the suspended catalyst. The CIP concentration was measured at 277 nm using a UH5300 UV-vis spectrometer (Hitachi), and the TBBPA concentration was measured at 209 nm using an LC 3000 HPLC system (Agilent Technology Co., Ltd., Santa Clara, CA, United States) with a Thermo Fisher C18 column. The mobile phase in the HPLC column was 0.1 % acetic acid aqueous solution (solution A) and methanol (solution B). The v/v ratio of solution A to B was 2:8. The flow rate was 1 ml/min, and the column temperature was 35°C.

The main active species generated in the photocatalytic process were investigated to determine the possible mechanism. Sodium oxalate (SO, 1 mmol/L), tert-butyl alcohol (TBA, 10 mmol/L), and benzoquinone (BQ, 1 mmol/L) were added to scavenge holes (h^+^), hydroxyl radicals (^⋅^OH), and superoxide radicals (^⋅^O_2_^–^), respectively (Bi et al., 2016).

All experiments were repeated three times, and the mean values were reported.

## 3. Results and discussion

### 3.1. Characterization of photocatalysts

#### 3.1.1. Crystal structures and morphologies

**FIGURE 1:**
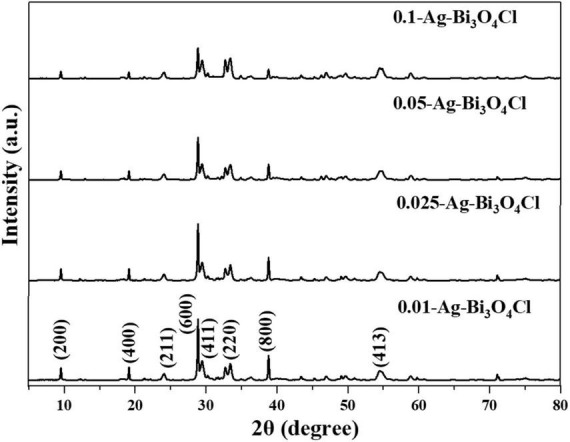
X-ray diffraction (XRD) patterns of Ag-Bi_3_O_4_Cl photocatalysts with different ratios.

The crystal structures of the catalysts were analyzed using X-ray diffraction (XRD). Figure 1 shows the XRD patterns of a series of Ag-Bi_3_O_4_Cl samples prepared with different Ag contents. The diffraction patterns of all Ag-Bi_3_O_4_Cl catalysts agree well with that of the monoclinic crystal Bi_3_O_4_Cl (JCPDS 36-0760). Compared with pure Bi_3_O_4_Cl, the peaks and lattice parameters of the Ag-Bi_3_O_4_Cl catalysts remain unchanged, confirming that Ag is deposited on the Bi_3_O_4_Cl surface rather than entering its lattice. In addition, the intensity ratio of the (600)/(411) peaks change slightly, suggesting that the Ag content can impact the preferred orientation and the crystallinity of Bi_3_O_4_Cl to a certain extent (Li J. et al., 2014). The diffraction peak of metallic Ag is not detected possibly because of its low content and good dispersion on the Bi_3_O_4_Cl surface. Moreover, no impurities or other phases are detected, indicating the high purity of the synthesized photocatalysts.

**FIGURE 2:**
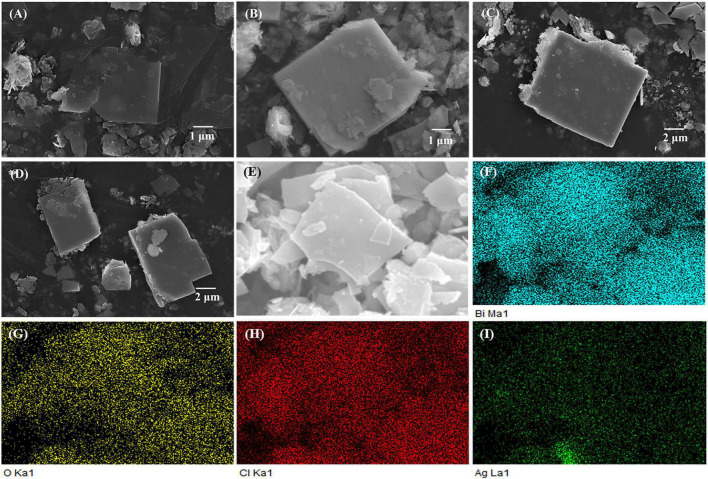
The SEM image and surface elements distribution of samples: **(A–D)** The SEM image of 0.01-Ag-Bi_3_O_4_Cl–0.1-Ag-Bi_3_O_4_Cl. **(E–I)** The surface elements distribution of 0.025-Ag-Bi_3_O_4_Cl.

Figures 2A–D show the scanning electron microscopy (SEM) images of the Ag-Bi_3_O_4_Cl plasmonic photocatalysts with different Ag loadings. All samples exhibit the nanoflake morphology identical to that of Bi_3_O_4_Cl, indicating that Ag addition does not change the morphology of Bi_3_O_4_Cl. However, the surfaces of the nanoflakes become rough with increasing Ag content, implying that their crystallinity has changed, consistent with the XRD results.

Figures 2E–I show the results of the energy-dispersive X-ray spectroscopy (EDS) surface elemental analysis of the 0.025-Ag-Bi_3_O_4_Cl. The spectra reveal that Bi, O, Cl, and Ag are uniformly distributed on the surface. This is consistent with the XRD results that Ag is not incorporated into the lattice of Bi_3_O_4_Cl but deposited on its surface. Moreover, the uniform elemental distribution facilitates the electron transfer in the photocatalytic degradation (Niu et al., 2020b).

**FIGURE 3:**
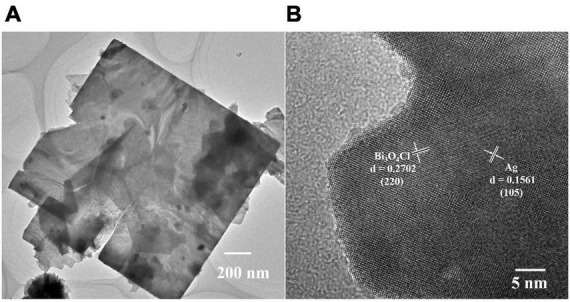
The TEM analysis of 0.025-Ag-Bi_3_O_4_Cl: **(A)** TEM image, **(B)** high resolution transmission electron microscopy (HRTEM) of the sample.

To further investigate the crystal structure of Ag-Bi_3_O_4_Cl, 0.025-Ag-Bi_3_O_4_Cl was selected as a representative sample for transmission electron microscopy (TEM) analysis. As shown in Figure 3A, it exhibits a nanoflake shape, consistent with the SEM images. In Figure 3B, the lattice spacings of 0.2702 and 0.1561 nm correspond to the (220) and (105) crystal planes of Bi_3_O_4_Cl and Ag, respectively. These results agree with the XRD and SEM observations and demonstrate that Ag is deposited on the surface of Bi_3_O_4_Cl and does not dissolve in the lattice.

The specific surface areas and porous structures of pure Bi_3_O_4_Cl and Ag-Bi_3_O_4_Cl with different Ag loadings are summarized in Table 1. As the Ag loading increases, the specific surface area and pore volume first increase and then decrease, with 0.025-Ag-Bi_3_O_4_Cl exhibiting the maximum specific surface area (19.971 m^2^/g) and highest pore volume (0.083 cm^3^/g). These results indicate that adding an appropriate amount of Ag to Bi_3_O_4_Cl can increase the surface area and pore volume, thereby enhancing the adsorption capacity. As the adsorption capacity increases, more pollutants can accumulate on the surface. The larger the specific surface area, the more active components are exposed, promoting the catalytic degradation of organic pollutants (Niu et al., 2020a).

**TABLE 1:** The BET surface areas, pore volume, and pore diameter of Bi_3_O_4_Cl, 0.025-Ag-Bi_3_O_4_Cl, and 0.05-Ag-Bi_3_O_4_Cl.

Sample	S_BET_ (m^2^/g)	Pore volume (cm^3^g^–1^)	Pore diameter (nm)
Bi_3_O_4_Cl	10.841	0.047	1.677
0.025-Ag-Bi_3_O_4_Cl	19.971	0.083	1.923
0.05-Ag-Bi_3_O_4_Cl	12.092	0.039	1.675

#### 3.1.2. Chemical compositions and valence state

**FIGURE 4:**
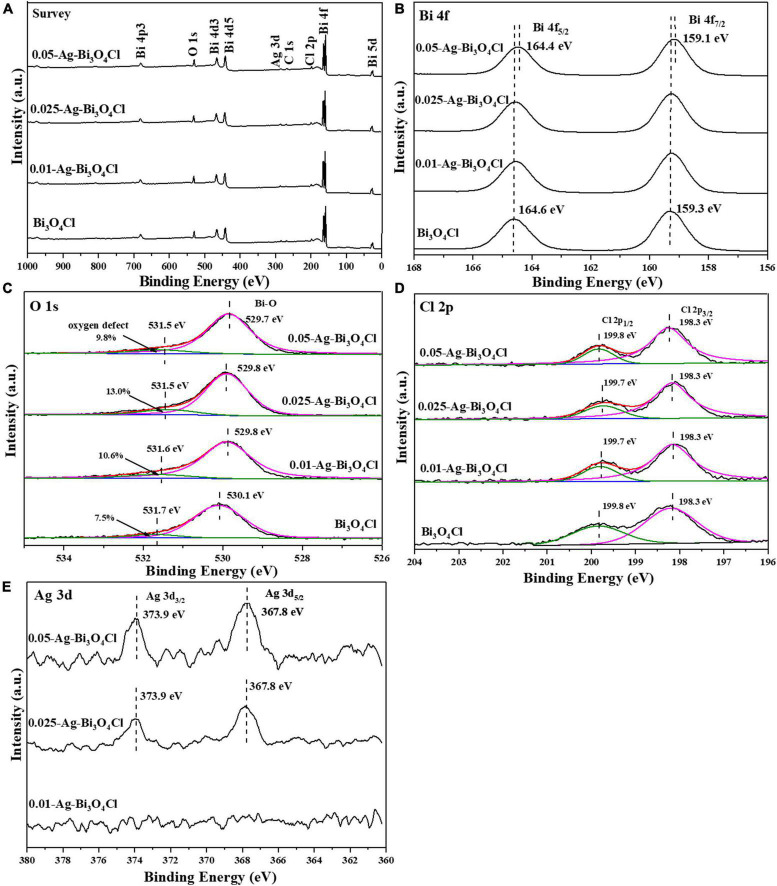
The X-ray photoelectron spectroscopy (XPS) spectra of photocatalysts: **(A)** survey; **(B)** Bi 4f; **(C)** O 1s; **(D)** Cl 2p; **(E)** Ag 3d.

Figure 4 shows the chemical compositions and valence states of Ag-Bi_3_O_4_Cl plasmon photocatalysts synthesized with different Ag ratios. As shown in Figure 4A, Bi, Cl, O, and C elements are found in all samples and no impurity peaks are observed. The characteristic peak of Ag is not detected for 0.01-Ag-Bi_3_O_4_Cl because of its low Ag content. With the increasing Ag loading, this peak emerges in the spectra of 0.025-Ag-Bi_3_O_4_Cl and 0.05-Ag-Bi_3_O_4_Cl.

Figure 4B shows the Bi 4f characteristic peaks of the samples. In pure Bi_3_O_4_Cl, two characteristic peaks at 159.3 eV and 164.6 eV were detected, corresponding to the Bi 4f_5/2_ and Bi 4f_7/2_ orbitals of the Bi trivalent chemical state, respectively (Zhang et al., 2020). In addition, with an increase in the Ag content, the diffraction peak shifted toward a low binding energy, indicating the presence of low-charged Bi ions at the external sites of the Bi_3_O_4_Cl nanosheet. Sun et al. (2019) reported similar observations, which were attributed to the introduction of Ag metal, leading to the generation of oxygen defects (Apelgren et al., 2019).

In Figure 4C, there are two characteristic peaks of O 1s at approximately 531.5–531.7 eV and 529.7–530.1 eV, corresponding to the O_2_^–^ in oxygen-deficient regions and lattice oxygen, respectively (Long et al., 2022b). Moreover, the oxygen defect content first increased and then decreased with increasing Ag content, and the presence of oxygen defects promoted electron-hole pairs separation in the photocatalysts (Zou et al., 2021). The presence of oxygen defects was further confirmed using electron spin resonance (ESR). In Supplementary Figure 1, the oxygen vacancies increase with the introduction of metallic Ag.

In Figure 4D, the two characteristic peaks of Bi_3_O_4_Cl are at approximately 199.8 and 198.3 eV, corresponding to Cl 2p_1/2_ and Cl 2p_3/2_, respectively (Wang et al., 2019). The characteristic peak of Cl 2p corresponding to Ag-Bi_3_O_4_Cl exhibits a trend of transition from a low binding energy to a high binding energy. The shift and transition of the Cl 2p characteristic peak in Ag-Bi_3_O_4_Cl may be due to the change in the Cl-O distance caused by oxygen defects; similar results have been observed in the study of Bi-BiOI (Sun et al., 2019).

In Figure 4E, the presence of elemental Ag in the 0.025-Ag-Bi_3_O_4_Cl and 0.05-Ag-Bi_3_O_4_Cl samples can be clearly observed, with two characteristic peaks of Ag 3d at approximately 373.9 and 367.8 eV, corresponding to Ag 3d_3/2_ and Ag 3d_5/2_, respectively (Phuruangrat et al., 2021). The intensities of the diffraction peaks tended to be increase with increasing Ag metal content. However, no characteristic Ag peak was observed in the 0.01-Ag-Bi_3_O_4_Cl sample, which may be because of the low content of Ag.

#### 3.1.3. Optical and electrochemical properties

**FIGURE 5:**
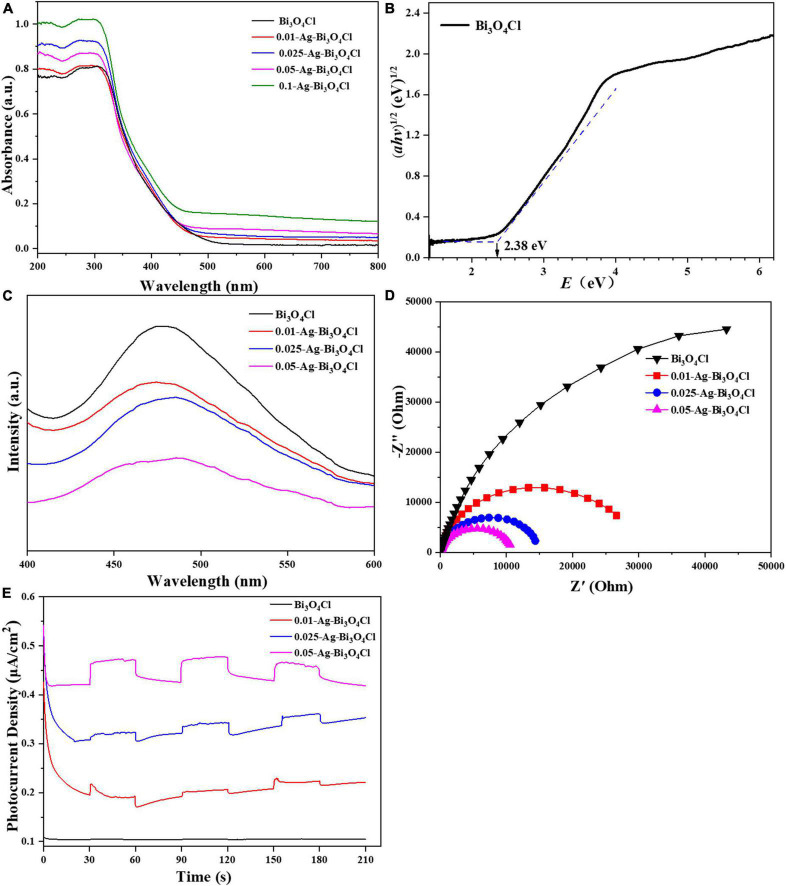
**(A)** UV–Vis diffuse reflection spectrum (DRS), **(B)** bandgap energies, **(C)** photoluminescence (PL) spectra, **(D)** EIS Nyquist plots, and **(E)** TPRs of the investigated catalysts.

The UV-vis diffuse reflectance spectroscopy results for the Bi_3_O_4_Cl and Ag-Bi_3_O_4_Cl plasmon photocatalysts are shown in Figure 5A. The maximum optical absorption wavelength of pure Bi_3_O_4_Cl is approximately 540 nm. The series of Ag-Bi_3_O_4_Cl plasmon photocatalysts exhibited absorption at all visible wavelengths from 500 to 800 nm, which increased with increasing Ag content. This result indicates that the Ag metal on the surface of Bi_3_O_4_Cl undergoes a plasmon resonance phenomenon under visible-light irradiation, which greatly increases its light absorption ability and broadens its light absorption range. From the plot of (*αhv*)^1/2^ versus energy (*hv*) (Liu et al., 2017), the band gap of the Bi_3_O_4_Cl photocatalyst was determined to be 2.38 eV (Figure 5B). These results indicate that the Ag-Bi_3_O_4_Cl photocatalyst has superior visible-light absorption ability, which is beneficial for the utilization of sunlight.

As shown in Figure 5C, the photoluminescence (PL) peak intensities of the Ag-Bi_3_O_4_Cl samples are weaker than those of Bi_3_O_4_Cl, indicating that the introduction of Ag reduced the electron-hole recombination rate of the photocatalyst. As the Ag content increased, the PL peak intensity of the samples gradually decreased. This is because excessive Ag metal hinders electron-hole pairs recombination and promotes carrier migration (Jiang et al., 2020).

The migration, conversion, and separation of electron-hole pairs in the Ag-Bi_3_O_4_Cl plasmon photocatalysts were evaluated using electrochemical impedance spectroscopy (EIS) and transient current response (TPR). A smaller arc radius of the Nyquist curve of the photocatalyst indicates faster charge transfer. The trend of the influence of the Ag content on the radius of the curved arc is consistent with that of the PL spectrum and also presents a gradually decreasing trend (Figure 5D). The photocurrent response of the photocatalyst (Figure 5E) exhibited a gradually increasing trend as the Ag content increased. This result is consistent with the PL and EIS results, indicating that the presence of Ag metal promotes the migration, conversion, and separation of the electron-hole pairs of Bi_3_O_4_Cl (Hong et al., 2023).

Based on the optical and electrochemical characteristics of the photocatalysts, it can be concluded that the Ag content significantly affects the photoelectric performance of Ag-Bi_3_O_4_Cl plasmon photocatalysts; with the increase in Ag content, the recombination of photogenerated electron-hole pairs is reduced, and the migration and separation capabilities of electrons and holes are enhanced, which is conducive to improving the performance of photocatalysts. However, in the photocatalytic oxidation of pollutants, the active substance that still plays the main role is Bi_3_O_4_Cl, and the presence of excessive Ag reduces the number of active sites of Bi_3_O_4_Cl, which may hinder the catalytic degradation of pollutants (Chen et al., 2014).

According to the above research, the morphology of Bi_3_O_4_Cl does not change after Ag deposition. Ag content affects the photoelectric performance of Bi_3_O_4_Cl, and an appropriate concentration of Ag can maximize the catalytic activity of Bi_3_O_4_Cl.

### 3.2. Performance of Ag-Bi_3_O_4_Cl photocatalyst toward EC degradation

#### 3.2.1. Applicability of Ag-Bi_3_O_4_Cl for CIP and TBBPA removal

As discussed in section “3.1. Characterization of photocatalysts,” Ag addition changes the physical and chemical properties of Bi_3_O_4_Cl, which in turn affects its catalytic activity. In this section, the applicability of Ag-Bi_3_O_4_Cl toward the removal of CIP and TBBPA (as the target contaminants) is verified by analyzing the degradation performance of Ag-Bi_3_O_4_Cl plasmonic photocatalysts with different Ag loadings.

**FIGURE 6:**
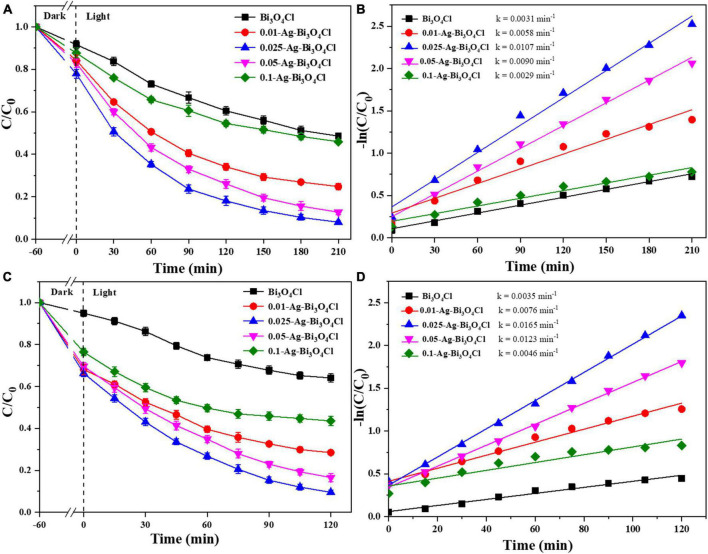
Ciprofloxacin (CIP) and TBBPA removal in different photocatalysts: **(A)** removal efficiency of CIP; **(B)** kinetics of CIP removal; **(C)** removal efficiency of TBBPA; **(D)** kinetics of TBBPA removal; conditions: the concentration of CIP and TBBPA = 10 mg/L, initial pH, catalyst dosage = 0.8 g/L.

As shown in Figures 6A, C, the adsorption capacities of the different photocatalysts for both CIP and TBBPA are ranked as follows: 0.025-Ag-Bi_3_O_4_Cl > 0.05-Ag-Bi_3_O_4_Cl > 0.01-Ag-Bi_3_O_4_Cl > 0.1-Ag-Bi_3_O_4_Cl > Bi_3_O_4_Cl. Moreover, a single Bi_3_O_4_Cl photocatalyst had a poor effect on the treatment of ECs. After 210 min, the removal rate of pure Bi_3_O_4_Cl for CIP was only 51.4%, whereas after 120 min, the removal rate of TBBPA was only 36.0%.

In comparison, the effect of the Ag-Bi_3_O_4_Cl plasmon photocatalysts on the treatment of CIP and TBBPA was better than that of single Bi_3_O_4_Cl, but the effect of samples prepared with different Ag contents was still different. For samples (0.01–0.1)-Ag-Bi_3_O_4_Cl, their photocatalytic degradation efficiencies for CIP were 75.2, 92.0, 87.3, and 54.1% after 210 min, respectively. After 120 min, their photocatalytic degradation efficiencies of TBBPA were 71.5, 90.5, 83.4, and 56.5%, respectively. As shown in Figures 6B, D, 0.025-Ag-Bi_3_O_4_Cl exhibits the highest degradation rates of CIP (0.0107 min^–1^) and TBBPA (0.0165 min^–1^). The degradation of CIP/TBBPA was 1.8/2.2, 1.2/1.3, 3.7/3.6, and 3.5/3.6 times higher than those of 0.01-Ag-Bi_3_O_4_Cl, 0.05-Ag-Bi_3_O_4_Cl, 0.1-Ag-Bi_3_O_4_Cl, and Bi_3_O_4_Cl, respectively.

These results indicate that the efficiency of the Ag-Bi_3_O_4_Cl plasmon photocatalyst prepared by adding an appropriate amount of Ag to treat ECs in the water is much higher than that of a single Bi_3_O_4_Cl photocatalyst. Moreover, during the preparation process, the variation in the Ag metal content affected the photocatalytic performance of Ag-Bi_3_O_4_Cl, which showed a trend of increasing and then decreasing with increasing Ag metal content.

Based on the characterization analysis results discussed in section “3.1. Characterization of photocatalysts,” the Ag content significantly affects the photoelectric performance of the Ag-Bi_3_O_4_Cl plasmon photocatalysts. The increase in Ag content reduces the recombination of photogenerated electron-hole pairs and enhances the migration and separation capabilities of electrons and holes, which is conducive to improving the performance of the photocatalyst. However, the active substance that plays the main role in the photocatalytic oxidation of pollutants is Bi_3_O_4_Cl. Excessive Ag metal content reduced the number of Bi_3_O_4_Cl active sites, which hindered the catalytic degradation of pollutants. Therefore, it is necessary to select an appropriate Ag content. The experiments described in this section show that 0.025-Ag-Bi_3_O_4_Cl is the optimal ratio.

#### 3.2.2. Effect of catalyst dosage and solution pH on EC removal

**FIGURE 7:**
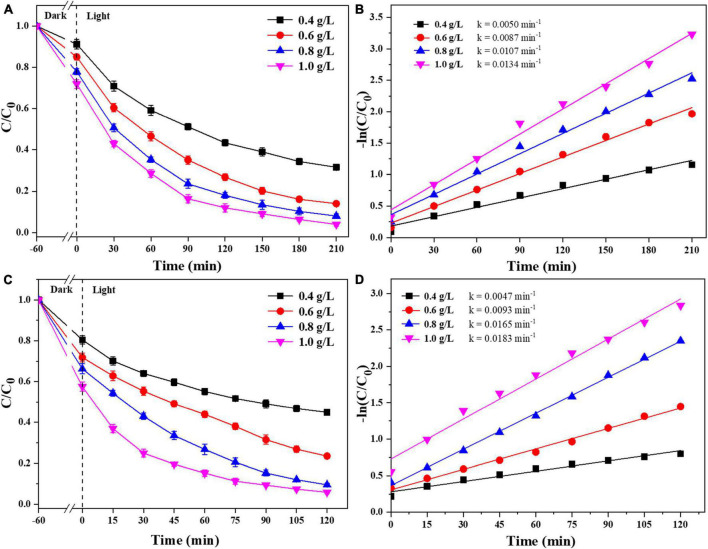
Removal efficiency of CIP and TBBPA by 0.025-Ag-Bi_3_O_4_Cl with different catalyst dosages: **(A)** removal efficiency of CIP; **(B)** kinetics of CIP removal; **(C)** removal efficiency of TBBPA; **(D)** kinetics of TBBPA removal.

We were selected that 0.025-Ag-Bi_3_O_4_Cl was used to study the effects of the photocatalyst dosage and reaction pH on the degradation of CIP and TBBPA. As shown in Figure 7, when the dosage of 0.025-Ag-Bi_3_O_4_Cl was increased from 0.4 to 1.0 g/L, the adsorption performance, removal efficiency, and reaction rate constant of CIP and TBBPA gradually increase with the increase of the catalyst dosage. This is because by adding more photocatalyst, more active sites were obtained, thus improving the adsorption and catalytic efficiency of CIP and TBBPA. Moreover, the dosage of 0.8 g/L of photocatalyst is sufficient to fully degrade CIP and TBBPA. Therefore, considering both cost and degradation efficiency, a catalyst dosage of 0.8 g/L was selected as the optimal dosage condition in this study, and 0.8 g/L is selected for the following experiments.

**FIGURE 8:**
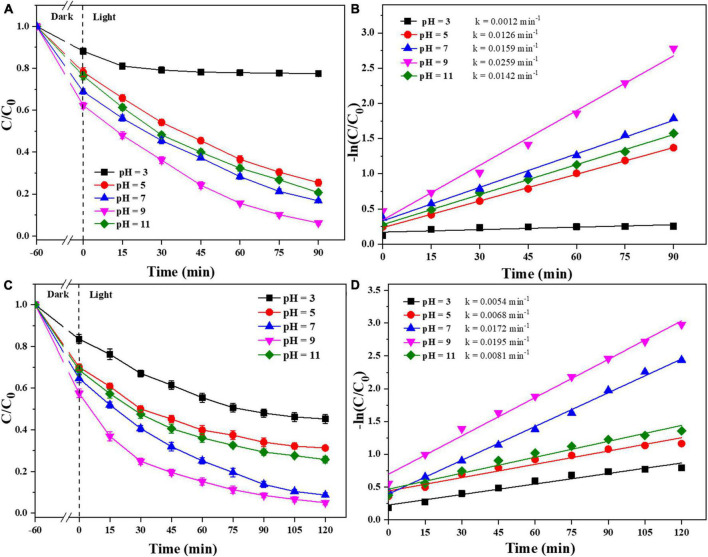
Removal efficiency of CIP and TBBPA by 0.025-Ag-Bi_3_O_4_Cl with different initial pH values of solution: **(A)** removal efficiency of CIP; **(B)** kinetics of CIP removal; **(C)** removal efficiency of TBBPA; **(D)** kinetics of TBBPA removal.

In addition, the effect of the initial pH of the pollutants on the photocatalyst degradation was studied. As shown in Figure 8, the photocatalysts were effectively degraded both CIP and TBBPA in weakly acidic and alkaline environments. When the initial pH of CIP and TBBPA is in a weak alkaline environment, 0.025-Ag-Bi_3_O_4_Cl exhibited the optimal adsorption and degradation effects (CIP: 93.8%; TBBPA: 94.9%) and the highest reaction rate constants (CIP: 0.02259 min^–1^; TBBPA: 0.0195 min^–1^). In contrast, the adsorption and treatment effects of the photocatalyst on pollutants are significantly reduced under strongly acidic conditions. Some studies have shown that the solubility of CIP is significantly affected by pH and can form cationic, anionic, or amphoteric species, which mainly depends on its different pK_*a*_ (6.1 and 8.7) (Mao et al., 2016). Among them, amphoteric CIP ions have the lowest solubility and highest hydrophobicity, which results in a higher adsorption of CIP on the photocatalyst when the pH is between 6.1 and 8.7, thus indirectly improving the reaction rate. When the pH is higher than the TBBPA pK_*a*_ (∼7.4), the degradation and debromination rates of TBBPA also increase sharply with an increase in pH, which contributes to easier degradation of TBBPA (Han et al., 2016). This also indicates that the Ag-Bi_3_O_4_Cl plasmon photocatalyst can adsorb and remove CIP and TBBPA in both weakly acidic and alkaline environments, which is a good practical application.

#### 3.2.3. Reusability and stability of Ag-Bi_3_O_4_Cl

**FIGURE 9:**
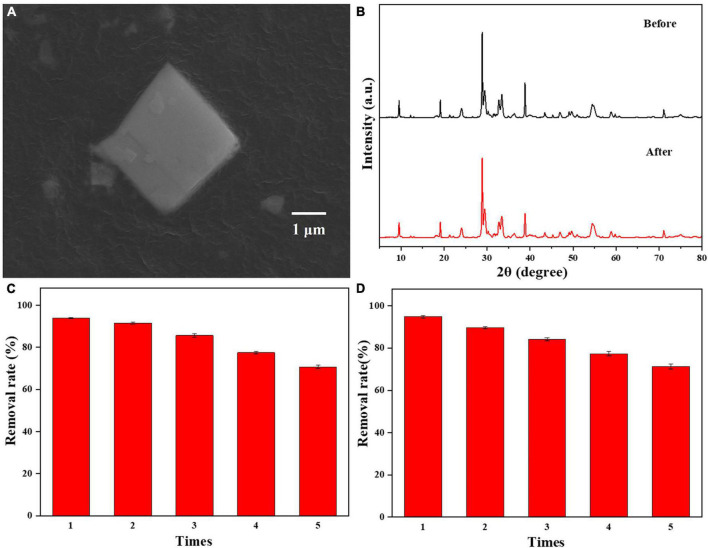
**(A)** SEM images of 0.025-Ag-Bi_3_O_4_Cl after use; **(B)** the XRD patterns of 0.025-Ag-Bi_3_O_4_Cl before and after use; reusability of 0.025-Ag-Bi_3_O_4_Cl in **(C)** CIP and **(D)** TBBPA degradation.

A reusability test of 0.025-Ag-Bi_3_O_4_Cl was performed; the results are shown in Figure 9. In Figure 9A, the SEM image of 0.025-Ag-Bi_3_O_4_Cl shows that its morphology did not change significantly before and after the reaction (Figure 2). Bi_3_O_4_Cl as the substrate is well preserved, and no Ag aggregation is observed on the photocatalyst surface. Figure 9B shows the XRD patterns of 0.025-Ag-Bi_3_O_4_Cl before and after the reaction, wherein the diffraction peaks are in the same position and the intensities remain unchanged, indicating that the photocatalyst has good stability.

Figures 9C, D show the photocatalytic degradation results of 0.025-Ag-Bi_3_O_4_Cl in five cycles. The CIP and TBBPA removal rates reach 70.6 and 71.2%, respectively, after five utilization cycles, suggesting the good reusability of the photocatalyst.

### 3.3. Photocatalytic mechanisms

#### 3.3.1. Radical quenching experiments and total organic carbon removal

**FIGURE 10:**
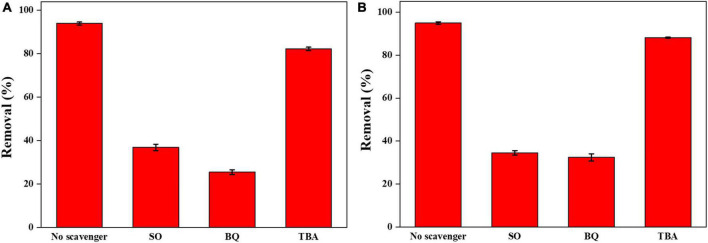
**(A)** Effects of various scavengers on the photocatalytic degradation of CIP; **(B)** effects of various scavengers on the photocatalytic degradation of TBBPA.

To explore the reaction mechanism of the Ag-Bi_3_O_4_Cl plasmonic photocatalyst system, radical-quenching experiments were conducted on the free radicals generated in the catalytic system (Figure 10).

The addition of TBA, as a quencher of ^⋅^OH, had only a slight effect on the 0.025-Ag-Bi_3_O_4_Cl photocatalytic degradation of CIP and TBBPA, indicating that ^⋅^OH did not play a decisive role in the photocatalytic degradation of CIP and TBBPA (Jiang et al., 2018).

The addition of SO, as a quencher of h^+^, significantly inhibited the 0.025-Ag-Bi_3_O_4_Cl photocatalytic degradation of both CIP and TBBPA. This result indicated that h^+^ was the active species that played a dominant role in the photocatalytic degradation of CIP and TBBPA. This is because of the high valence-band (VB) position of Ag-Bi_3_O_4_Cl, and h^+^ can directly oxidize organic pollutants (Long et al., 2022a).

The addition of BQ, as a quencher of ^⋅^O_2_^–^, also had a significant inhibitory effect on the photocatalytic degradation of CIP and TBBPA by 0.025-Ag Bi_3_O_4_Cl. This is due to the plasmon resonance phenomenon of Ag metal elements with light illumination, which greatly promotes the separation of electron-hole pairs and the generation of ^⋅^O_2_^–^, which can effectively oxidize and decompose pollutants in water (Li F. et al., 2014).

From the above results, it is clear that photo-generated holes (h^+^) and superoxide radicals (^⋅^O_2_^–^) play the dominant role in the photocatalytic degradation of CIP and TBBPA.

The photocatalytic degradation of CIP and TBBPA using total organic carbon (TOC) was investigated. After 210 min (CIP) and 120 min (TBBPA) of visible light irradiation, the removal rates of TOC reached 40.7 and 47.2%, respectively. Further removal of these intermediates was achieved by increasing the reaction time. These results indicate that the Ag-Bi_3_O_4_Cl plasmon photocatalysts are effective for the treatment of ECs and exhibit good pollutant mineralization ability.

#### 3.3.2. Degradation pathways of CIP and biotoxicity analysis of intermediate products

**FIGURE 11:**
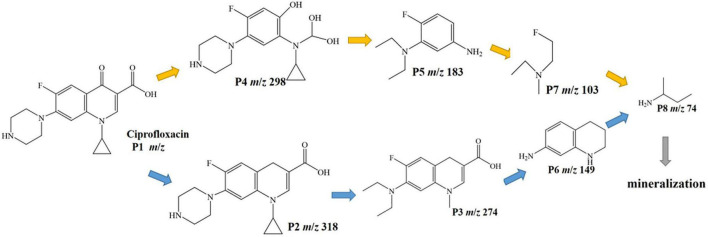
Suggested photocatalytic degradation pathways of CIP.

The intermediates of CIP photodegradation on 0.025-Ag Bi_3_O_4_Cl were detected by LC-MS (Supplementary Figure 2 and Supplementary Table 1). Based on these fragments and previous reports, two different degradation pathways were inferred in Figure 11 (Dewitte et al., 2008; Gao et al., 2021). In pathway I, CIP was attacked by the strong oxidizing radical and deoxygenated to produce the product P2 (m/z = 318). Next, the piperazine ring was broken and the ternary ring was further damaged by oxidation to produce products P3 (m/z = 274), P6 (m/z = 149), and P8 (m/z = 74). In pathway II, CIP was attacked by the oxide species and substituted with hydroxyl groups to generate product P4 (m/z = 298). Next, the C-N bond of the piperazine ring was broken and the ternary ring is removed, producing intermediate P5 (m/z = 183) and through a series of cleavage processes intermediates P7 (m/z = 103), P8 (m/z = 74) were generated. Finally, the above intermediate products could be decomposed into CO_2_ and H_2_O.

The biological toxicity of CIP degradation products was analyzed using EPI-ECOSAR software. There were two possible pathways for the degradation of CIP by 0.025-Ag-Bi_3_O_4_Cl plasmon photocatalysts, namely CIP-P2-P3-P6-P8 and CIP-P4-P5-P7-P8. As shown in Supplementary Table 2, the biotoxicity of the intermediate products of CIP showed a trend of first significantly increasing in toxicity and then gradually decreasing. The biotoxicity of some intermediate products (P3 and P5) were much higher than that of CIP. Therefore, more attention should be paid to the detection of biotoxicity of degradation products in the treatment of ECs.

#### 3.3.3. Possible mechanism of CIP and TBBPA removal over Ag-Bi_3_O_4_Cl

**FIGURE 12:**
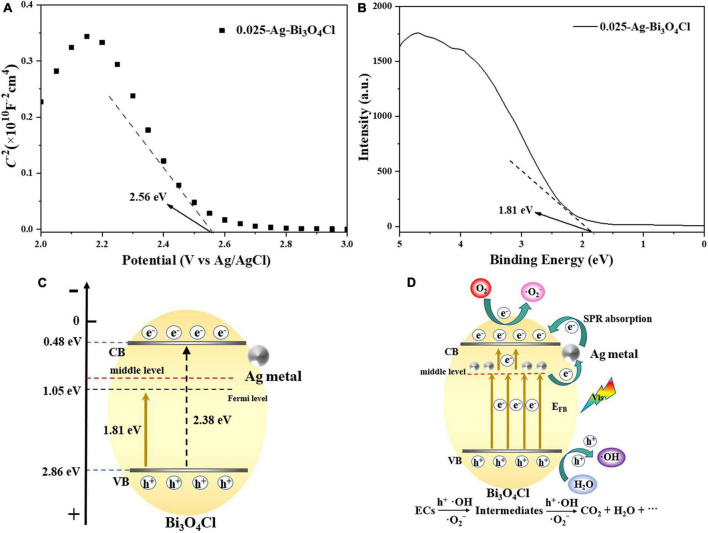
**(A)** Mott–Schottky plots of 0.025-Ag-Bi_3_O_4_Cl; **(B)** valence band (VB) XPS spectra of 0.025-Ag-Bi_3_O_4_Cl; **(C)** structural shifts of 0.025-Ag-Bi_3_O_4_Cl plasmon photocatalyst; **(D)** possible mechanism of Ag-Bi_3_O_4_Cl degrading ECs.

To illustrate the mechanism of the Ag metal-modified Bi_3_O_4_Cl at the electronic structure level, the flat band potential (*E*_*FB*_) of 0.025-Ag-Bi_3_O_4_Cl was measured using Mott–Schottky plots. As shown in Figure 12A, the negative slope of the tangent line in the Mott–Schottky plot of 0.025-Ag Bi_3_O_4_Cl, which is a typical p-type semiconductor (Abe et al., 2015), indicates that the introduction of Ag metal did not change the semiconductor type of Bi_3_O_4_Cl. The flat band potential (*E*_*FB*_) of 0.025-Ag Bi_3_O_4_Cl was 2.56 eV [2.76 eV versus normal hydrogen electrode (NHE)]. The *E*_*VB*_ position of the p-type semiconductor is close to its *E*_*FB*_ potential, which is approximately positive for *E*_*FB*_ (0.1 eV) (Zheng and Lei, 2018). From this, it can be seen that in this work, the corresponding potential of *E*_*VB*_ of 0.025-Ag Bi_3_O_4_Cl is approximately 2.86 eV versus NHE. Figure 12B shows the X-ray photoelectron spectroscopy (XPS)-VB spectrum of 0.025-Ag-Bi_3_O_4_Cl, and the potential difference between VB of 0.025-Ag-Bi_3_O_4_Cl and its Fermi energy level is 1.81 eV as can be seen from the figure. Moreover, according to the formula *E*_*CB*_ = *E*_*VB*_ - *E*_*g*_ (Long et al., 2020c) and UV-vis results (The bandwidth *E*_*g*_ for Bi_3_O_4_Cl is 2.38 eV), the *E*_*CB*_ and *E*_*VB*_ value of 0.025-Ag-Bi_3_O_4_Cl were calculated as 0.48 and 2.86 eV, respectively (Figure 12C).

Based on the above analysis and related literature reports (Ma et al., 2015), potential photocatalytic mechanisms involving electron migration pathways and the catalytic degradation of pollutants by Ag-Bi_3_O_4_Cl plasmon photocatalysts were proposed (Figure 12D). During the photocatalytic reaction, the charge carriers in the VB of Bi_3_O_4_Cl were excited by visible light. When Ag metal is deposited on the surface of Bi_3_O_4_Cl, it increases the concentration of oxygen defects, resulting in the formation of an intermediate energy level in the semiconductors (Pan and Heagy, 2019). In addition, the plasmon resonance effect generated by the Ag metal under illumination greatly increased the light absorption ability of the photocatalyst (Shi et al., 2018). The coexistence of oxygen defects and Ag metals promotes electron transfer (Gao et al., 2014). Moreover, before Bi_3_O_4_Cl was modified, its electrons were directly excited from the VB to the conduction band (CB), making it prone to electron-hole pair recombination. In contrast, after plasmon modification, the electron migration path of the photocatalyst shifts to the intermediate energy level formed by Ag-Bi_3_O_4_Cl, where electrons from the VB in Bi_3_O_4_Cl are first excited, and then some of them are stored by Ag metal. Another part of the electron transitions from the Ag metal to the CB of Bi_3_O_4_Cl, which greatly hinders the probability of electron-hole recombination and can effectively improve the performance of the photocatalyst. Since the redox potential of O_2_/^⋅^O_2_^–^ is −0.33 eV, which is more negative than the CB position of 0.025-Ag-Bi_3_O_4_Cl (0.48 eV), it is theoretically impossible to generate ^⋅^O_2_^–^. However, due to the plasmon resonance effect of Ag metal, the electrons excited to the CB of Bi_3_O_4_Cl via Ag metal can react with O_2_, thus generating ^⋅^O_2_^–^ free radicals, which is consistent with the phenomenon reported by Jiang et al. (2018). The carrier charge separation led to the generation of a large number of h^+^ ions in the VB of Ag-Bi_3_O_4_Cl. And the h^+^ can also react with water to generate strong oxidizing radicals ^⋅^OH. Finally, the generated h^+^, ^⋅^O_2_^–^, and ^⋅^OH have strong oxidation ability, gradually degrading emerging contaminants into small molecule organic compounds, CO_2_, and H_2_O.

The potential reaction process for the Ag-Bi_3_O_4_Cl photocatalytic degradation of the ECs is shown in Eqs. (1–7).


(1) $$\mathrm{Ag}-{\text{Bi}}_{3}\,{\text{O}}_{4}\,{\text{Cl}}_{\left(\text{s}\right)}+\text{hv}\to {\text{e}}_{\text{CB}}^{-}+{\text{h}}_{\text{VB}}^{+}$$

(2) ECs+hv→ECs*+eCB-

(3) $${\text{O}}_{2}+{\text{e}}_{\text{CB}}^{-}\to \cdot {\text{O}}_{2}^{-}$$

(4) $${\text{H}}_{2}\text{O}+{\text{h}}_{\text{VB}}^{+}\to \cdot \text{OH}+{\text{OH}}^{-}$$

(5) $${\text{OH}}^{-}+{\text{h}}_{\text{VB}}^{+}\to \cdot \text{OH}$$

(6) $${\text{h}}_{\text{VB}}^{+}+\text{ECs}\to \text{intermediates}\to {\text{CO}}_{2}+{\text{H}}_{2}\,\text{O}$$

(7) $$\cdot {\text{O}}_{2}^{-}+\text{ECs}\to \text{intermediates}\to {\text{CO}}_{2}+{\text{H}}_{2}\text{O}$$

## 4. Conclusion

In this study, a series of Ag-Bi_3_O_4_Cl plasmonic photocatalysts were synthesized using a photodeposition method and characterized in detail. The introduction of Ag does not alter the morphology of Bi_3_O_4_Cl. With an increase in the Ag content, the oxygen defects on the photocatalyst surface first increase and then decrease. An appropriate Ag content of Ag-Bi_3_O_4_Cl can effectively enhance the photoelectric performance. The presence of Ag and oxygen defects promote the electron transfer in the photocatalyst. Under the optimal conditions (Ag content: 0.025, catalyst dosage: 0.8 g/L, and solution pH: 9), the Ag-Bi_3_O_4_Cl plasmonic photocatalyst can remove 93.8% of CIP and 94.9% of TBBPA, under visible light. Photogenerated holes (h^+^) and superoxide radicals (^⋅^O_2_^–^) are strongly oxidative radicals that play major roles in the photocatalytic reactions. The CIP degradation pathway was analyzed; the biological toxicity of CIP degradation products showed a trend of first increasing and then decreasing. In this study, a green, and stable plasmonic photocatalyst was developed and applied for the efficient removal of ECs from water and the biological toxicity of the degradation products was analyzed.

## Data availability statement

The original contributions presented in this study are included in the article/Supplementary material, further inquiries can be directed to the corresponding authors.

## Author contributions

ZL: conceptualization, methodology, and writing—original draft preparation. TG: data curation, investigation, and validation. CC: data curation and validation. GZ: supervision, visualization, investigation, writing—review, and editing. JZ: visualization, writing—review and editing, and funding acquisition. All authors contributed to the article and approved the submitted version.
