# Supplementary material for: Preparation and application of Ag plasmon Bi3O4Cl photocatalyst for removal of emerging contaminants under visible light
Source: Front Microbiol. 2023 Jun 9;14:1210790. doi: 10.3389/fmicb.2023.1210790 (PMC10289886; doi:10.3389/fmicb.2023.1210790)
Supplement: Supplementary file 1 [file Data_Sheet_1.DOCX]

**Preparation and application of Ag plasmon Bi_3_O_4_Cl photocatalyst for removal of emerging contaminants under visible light**

Zeqing Long^1^, Tingting Guo^2^, Chao Chen^2^, Guangming Zhang^3*^, Jia Zhu^4#^

1. Department of Public Health and Preventive Medicine, Changzhi Medical College, Changzhi, 046000, China.

2. Heping Hospital Affiliated to Changzhi Medical College, Changzhi, 046000, China.

3. School of Energy & Environmental Engineering, Hebei University of Technology, Tianjin, 300130, China.

4. School of Materials and Environmental Engineering, Shenzhen Polytechnic, Shenzhen 518055, China.

* Corresponding author: Guangming Zhang, Address: School of Energy & Environmental Engineering, Hebei University of Technology, Tianjin, 300130, China. Tel: +86 13520956445, E-mail: [2020017@hebut.edu.cn](mailto:2020017@hebut.edu.cn)

# Corresponding author: Jia Zhu, School of Materials and Environmental Engineering, Shenzhen Polytechnic, Shenzhen 518055, China, E-mail: [zhujia@szpt.edu.cn](mailto:zhujia@szpt.edu.cn)

Tingting Guo co-first author.

## 2.1 Materials

Bismuth nitrate pentahydrate, benzoquinone, CIP, and TBBPA were purchased from Macklin Biochemical Co., Ltd. (Shanghai, China). Potassium chloride, sodium oxalate, and sodium hydroxide were purchased from Sinopharm Chemical Reagent Co., Ltd. (China). Ethanol and tert-butyl alcohol were obtained from Aladdin Co., Ltd. (China), and the standard Ag sample (1 mg/mL) was purchased from the National Institute of Metrology, China.

## 2.3 Characterization

The crystal structures of photocatalysts were characterized by powder X-ray diffraction (XRD) in the 2θ range of 5–80°, using an X-ray diffractometer (Rigaku D/max-2200/PC, Japan) at 40 kV and 40 mA with Cu Kα (λ = 0.15405 nm) radiation. The morphology and microstructures of photocatalysts were characterized by scanning electron microscopy (SEM) and high-resolution transmission electron microscopy (HRTEM); SEM was performed using a Merlin (Zeiss, German) scanning electron microscope and HRTEM was conducted using a JEM2100 (Electronics Co., Ltd. Japan) microscope. Surface elemental chemical states and oxygen deficiencies of photocatalysts were characterized by X-ray photoelectron spectroscopy (XPS) using a 250Xi photoelectron spectrometer (Semmer, UK). To examine the light absorption range of the photocatalyst, ultraviolet-visible (UV-vis) diffuse reflection spectra (DRS) of photocatalysts were conducted on a UV-vis spectrophotometer (UV-2700, Shimadzu, Japan) with BaSO_4_ as a reference. Charge separation of the photocatalysts was characterized by photoluminescence spectroscopy (PL) using a diode array spectrometer module (UV-20, Horiba, France) with a 270 nm ultraviolet monochromatic lamp irradiation. The photocurrent, electrochemical impedance spectra (EIS), and Mott–Schottky analyses were conducted by CHI 760 E electrochemical workstations. The electrochemical analyses were conducted in a three-electrode quartz cell having 0.5 mol/L Na_2_SO_4_ electrolyte solution. A platinum wire was used as the counter electrode, saturated Ag/AgCl electrode was used as the reference electrode, and the sample-coated fluorine doped tin oxide (FTO) electrode was used as the working electrode. The test of the reactive radicals trapped with electron spin resonance (ESR) was carried out using a Bruker A300 (Bruker, German). Typically, 10 mg of photocatalyst was dissolved in 500 μL of methanol (DMPO-·O_2_^-^), after which 45 μL DMPO was added, followed by ultrasonic dispersion for 10 min.

**Figure S1**


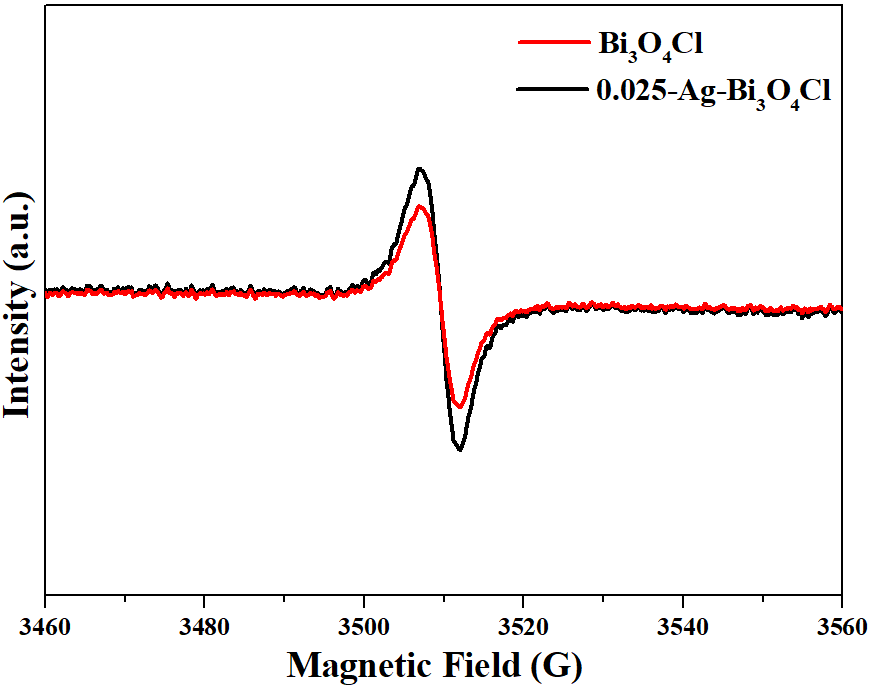


Figure S1 ESR spectra of 0.025-Ag-Bi_3_O_4_Cl and Bi_3_O_4_Cl.

**Figure S2**

**P1**

**P2 and P3**

**P4**

**P5**

**P6**

**P7**

**P8**

Figure S2. MS spectra of CIP and possible intermediates.

**Table S1**

Table S1 Ciprofloxacin Degradation Products

| CIP degradation products | m/z [M+H]^+^ | molecular formula | structural formula |
| --- | --- | --- | --- |
| CIP-P1 | 332 | C_17_H_18_FN_3_O_3_ | 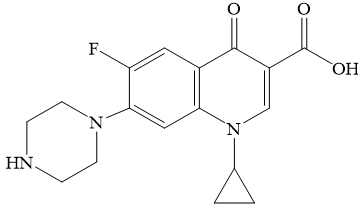 |
| P2 | 318 | C_17_H_20_FN_3_O_2_ | 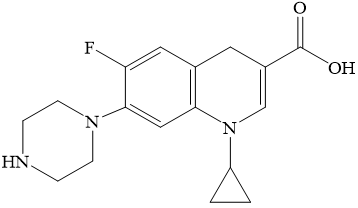 |
| P3 | 274 | C_15_H_16_FN_2_O_2_ |  |
| P4 | 298 | C_14_H_20_FN_3_O_3_ |  |
| P5 | 183 | C_10_H_15_N_2_F |  |
| P6 | 149 | C_9_H_12_N_2_ |  |
| P7 | 103 | C_5_H_11_NF |  |
| P8 | 74 | C_4_H_11_N |  |

**Table S2**

Table S2. Toxicity analysis of intermediates degraded by CIP

| CIP degradation products | structural formula | species | Time (h) | LC50/EC50  (mg/L) |
| --- | --- | --- | --- | --- |
| CIP | 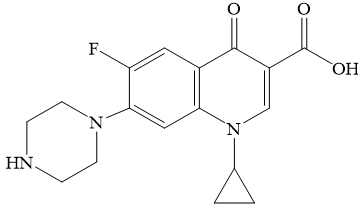 | Fish | 96 | 17053.15 |
|  |  | Daphnid | 48 | 8049.57 |
|  |  | Green Algae | 96 | 2793.44 |
| P2 | 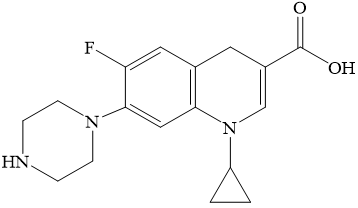 | Fish | 96 | 1898.18 |
|  |  | Daphnid | 48 | 986.37 |
|  |  | Green Algae | 96 | 509.30 |
| P3 |  | Fish | 96 | 92.26 |
|  |  | Daphnid | 48 | 60.46 |
|  |  | Green Algae | 96 | 81.46 |
| P4 |  | Fish | 96 | 4984.60 |
|  |  | Daphnid | 48 | 2473.71 |
|  |  | Green Algae | 96 | 1055.96 |
| P5 |  | Fish | 96 | 60.74 |
|  |  | Daphnid | 48 | 35.91 |
|  |  | Green Algae | 96 | 31.60 |
| P6 |  | Fish | 96 | 260.44 |
|  |  | Daphnid | 48 | 142.94 |
|  |  | Green Algae | 96 | 92.53 |
| P7 |  | Fish | 96 | 739.80 |
|  |  | Daphnid | 48 | 381.65 |
|  |  | Green Algae | 96 | 191.24 |
| P8 |  | Fish | 96 | 778.44 |
|  |  | Daphnid | 48 | 394.23 |
|  |  | Green Algae | 96 | 183.01 |
